# Ab Initio Molecular Dynamics Study of Quadrupolar Spin Relaxation in an Ionic Liquid

**DOI:** 10.1002/jcc.70311

**Published:** 2026-01-24

**Authors:** Luciano N. Vidal, Lucas C. Ducati, Jochen Autschbach

**Affiliations:** ^1^ Departamento de Química e Biologia Universidade Tecnológica Federal do Paraná Curitiba Paraná Brazil; ^2^ Department of Fundamental Chemistry Institute of Chemistry University of São Paulo São Paulo Brazil; ^3^ Department of Chemistry University at Buffalo, State University of New York Buffalo New York USA

## Abstract

This work implements a methodology for studying quadrupolar nuclear spin relaxation in ionic liquids. The dynamic behavior of the ions in the liquid is described by ab initio molecular dynamics (aiMD) with forces obtained from density functional theory (DFT) calculations with periodic boundary conditions and a non‐hybrid functional. The electric field gradient (EFG) driving the quadrupolar relaxation was calculated with free boundary conditions, using clusters that contained the ion of interest surrounded by two coordination shells treated quantum mechanically and augmented with a solvation model. Tests showed that EFG calculations using only the first coordination shell, containing five nearest neighbors, also provide a suitable model, because the relaxation rates differ by no more than 4% from the results from the two‐shell solvation. The results of this study show that the 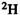 relaxation of the deuterated ethylammonium nitrate (${\text{EtND}}_{3}{\mathrm{NO}}_{3}$) occurs within the extreme narrowing regime for a spectrometer magnetic field $B_{0}=11.7$ T and is therefore characterized by the ensemble variance of the EFG and the correlation time associated with the EFG autocorrelation function. The quadrupolar relaxation of ${\text{EtND}}_{3}{\mathrm{NO}}_{3}$ demanded molecular dynamics production times longer than 330 ps and averaging over multiple ions, as well as independent trajectories to get suitably converged relaxation rates. The calculated 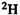 relaxation rate is $1/T_{1}=13.3\pm 1.0$ Hz, about 60% above the rate reported experimentally. However, the approach utilized in the present study has an accuracy similar to, or better than, what has been previously reported for systems involving non‐ionic solvents that required simulations of 100 ps duration or less.

## Graphical abstract

This study presents a methodology for describing the relaxation of nuclear spins (with I > 1/2), applicable to ionic liquids of a general nature. Based on ab initio molecular dynamics, we demonstrate the importance of explicit solvation in calculating the relaxation times of the quadrupolar mechanism.

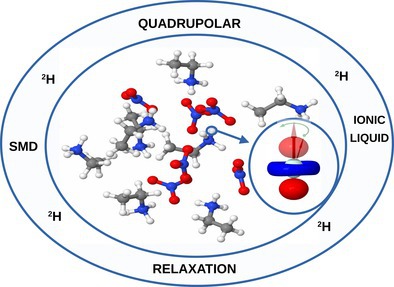

## 1 Introduction

Nuclear magnetic resonance (NMR) spectroscopy is a versatile and widely used technique to gather structural and conformational information in organic and inorganic systems by analyzing chemical shifts, J‐couplings, and quadrupolar interactions. Furthermore, NMR relaxation times provide crucial information on the dynamics of liquids and solutions, where the technique can probe motion at time scales varying from milliseconds to nanoseconds, for example, through fast field cycling NMR relaxometry [1]. Decades ago, the theoretical basis for nuclear spin relaxation was established [2, 3, 4, 5, 6, 7]. The time required for an ensemble of spins in an external magnetic field to return to equilibrium from a non‐equilibrium state created by a radio frequency pulse depends on the spin–lattice and spin–spin interactions. Several mechanisms contribute to relaxation in diamagnetic samples, including direct (dipolar) and indirect (electron‐mediated) magnetic nuclear spin–spin coupling, chemical shift anisotropy, chemical exchange, and spin‐rotation coupling [7, 8].

Nuclides with spin I>12 are not spherical, having a prolate or oblate shape, which goes along with an electric quadrupole moment [9, 10]. The spin relaxation rates of quadrupolar nuclides tend to be dominated by the interaction of the nuclear electric quadrupole with the electric field gradient (EFG) created by the distribution of charges elsewhere in the system, viz., by other atomic nuclei and the electron charge density. When an atom is in a site of high symmetry, (e.g., octahedral or tetrahedral), the EFG at the nucleus vanishes such that the quadrupolar interaction is suppressed. In solid‐state NMR, the quadrupolar interaction leads to characteristic NMR peak shapes, which is a great source of information about a system's electronic structure in addition to the chemical shift (and J‐couplings) [11]. In solution, when present, the quadrupolar interaction may cause substantial line broadening due to fast nuclear spin relaxation [7]. At the same time, the relaxation encodes important information about the liquid's dynamics. The present work focuses on the calculation of quadrupolar relaxation in an ionic liquid from first principles.

The key ingredient in computing a relaxation rate from the quadrupolar interaction is the time evolution of the EFG at an analyte nuclide in the form of an autocorrelation function (ACF) [8]. This information is accessible, for instance, via molecular dynamics (MD) simulations and on‐the‐fly or subsequent calculations of the EFG tensor and the resulting quadrupolar interaction at the nuclei of interest along the MD trajectories. Examples of such an approach, either from classical (force‐field based) [12, 13, 14] or ab initio molecular dynamics (usually with forces coming from density functional theory (DFT)) [15, 16, 17], are available in the literature.

The EFG itself can be computed from wave‐function or density‐based electronic structure methods [18, 19]. For systems where relaxation arises from solvated atoms or monoatomic ions, the Sternheimer approximation [20, 21] is often used to save computational resources related to the EFG calculation [22, 23, 24, 25, 26]. However, calculations of EFG tensors by DFT, for instance, are quite fast and do not invoke the rather severe approximations inherent in the Sternheimer model [27]. Therefore, a first‐principles theoretical approach such as DFT is preferable for obtaining the requisite EFG tensors. It has also been shown recently that machine‐learning models are able to speed up first‐principles theory‐based EFG calculations substantially, without leading to a deterioration of the resulting calculated relaxation rates [28].

An important type of non‐flammable and effectively non‐volatile solvent used as an alternative to traditional organic solvents is known as an *ionic liquid* (IL). ILs are molten salts formed by pairs of ions composed of organic and/or inorganic units. Commonly present cations are imidazolium, triazolium, phosphonium, pyridinium, pyrrolidinium, and alkylammonium. Anions include halide, nitrate, perchlorate, sulfate, nitrite, hexafluorophosphate, tetrafluoroborate, azide, and various organic anions such as triflate, benzoate, sulfacetamide, alkylsulfates, alkylcarbonates, and organic carboxylates [29, 30]. By definition, an IL must be liquid at pressures around 1 atm and temperatures below $100^{{}^{\circ}}C$. ILs have found numerous applications, including organic synthesis, photochemistry, food science, and power generation [13]. Because ILs have organic components, molecular designs can be tailored toward specific applications. However, this renders knowledge about the structure and dynamics of ILs and their interaction with other materials essential. Experimental techniques based on NMR have been used widely in studying ILs, such as self‐diffusion measurements, relaxometry, and two‐dimensional NMR. Relaxation processes of quadrupolar nuclei have been extensively studied in ILs, either with quadrupolar nuclides of high natural abundance (e.g., ^11^B, ^14^N, ^35^Cl, ^81^Br) [31, 32, 33, 34], or via isotopic substitution (e.g., ^2^H or ^17^O) [35, 36, 37, 38, 39, 40, 41, 42].

An important group of ILs is that of *protic ionic liquids* (PILs), formed by combining a Brønsted acid such as ammonium or alkylimidazolium cations with a Brønsted base such as carboxylate or bis(perfluoroethylsulfonyl)imide anions. In PILs, a proton can be transferred from the acid to the base or shared between them, leading to an additional structuring of the IL via the formation of hydrogen bonds. PILs have been used in organic synthesis, chromatography, biological applications, explosives, and acting as industrial lubricants [43]. The thermal stability, the conductivity of the acidic proton under anhydrous conditions, and a wide electrochemical window render PILs useful for applications such as fuel cell electrolytes [44]. A curious aspect observed in PILs is the formation of ionic aggregates of the same charge, where the strength of the hydrogen bond overcomes the Coulomb repulsion, generating cationic [45, 46] or anionic clusters [47]. Studies that seek to elucidate structural and dynamic aspects of PILs and the various types of hydrogen bonds often combine NMR techniques with infrared absorption measurements [37], neutron diffraction [48], and MD simulations [47]. The latter are considered well‐established for ILs and are in widespread use, either based on force fields or first‐principles methods to generate the forces [49, 50].

The Sternheimer approximation was evaluated for an ionic liquid in a study of the quadrupolar relaxation of ^23^Na^+^ ions in 1‐ethyl 3‐methylimidazolium tetrafluoroborate, [Im21][${\mathrm{BF}}_{4}$] [13]. Due to the slow dynamics of the IL, simulations ranging from 25 to 50 ns were necessary to adequately capture the asymptotic decay of the EFG ACFs for the entire range of temperatures considered (300−500 K). The study concluded that the fluctuations of the EFG, which determine the magnitude of the longitudinal relaxation rate, occur mainly due to the movement of the solvent around the cation, where, on average, the ${{\mathrm{BF}}_{4}}^{-}$ anions form a tetrahedral arrangement around sodium. Recently, Rumble et al. combined classical dynamics with the Sternheimer approximation to study the relaxation of ^23^Na^+^ in a mixture of water and [Im21][${\mathrm{BF}}_{4}$] at different proportions and temperatures, showing that the effect of composition on $T_{1}$ is related to changes between the inertial and diffusive relaxation regimes [51]. Other studies based on classical molecular dynamics of the quadrupolar relaxation of solvated species in ionic liquids exist [40, 52].

Considering the relevance of ILs in several areas of knowledge, as mentioned already, and the importance of a greater understanding of the dynamic behavior of these systems on a molecular scale, we assess herein a theoretical methodology to study the quadrupolar relaxation processes in this type of system based on DFT calculations of EFG tensors combined with DFT‐based MD. This type of dynamics is often dubbed “ab initio MD” (aiMD) because the forces are generated by first‐principles calculations, although we note that quantum nuclear effects are often neglected in aiMD—as in the present work—because of the high computational cost/benefit ratio when considering them. The availability of experimental data for the quadrupolar relaxation of deuterium in PILs [35, 36, 38, 39], together with the existence of several studies focusing on the structure of the PIL ethylammonium nitrate (EAN) [37, 53, 54, 55], motivated the choice of deuterated EAN (EAN‐3D) for the development of a relaxation study based on aiMD. The simulations were based on a non‐hybrid generalized gradient approximation (GGA) exchange‐correlation functional to be able to access several hundred picoseconds of dynamics with multiple independent trajectories. EFG tensors were calculated using a range‐separated hybrid functional parametrized to include a correction for non‐covalent (dispersion) interactions. The remainder of the article includes a synopsis of the underlying relaxation theory and its implementation in a newly developed code, computational details regarding the aiMD and the process of extracting clusters containing the deuterated ethylammonium cation and its first two shells of neighboring ions, details about the electronic structure calculations of the EFG, and some technical details of the code implementation. Subsequently, we assess, analyze, and discuss the results obtained for the EAN‐3D ionic liquid.

## 2 Theory

Key elements of the theory are outlined in this section to render the article reasonably self‐contained. The computational treatment of NMR relaxation rates is based on the relaxation theory of Bloch, Redfield, and others [7, 8, 56]. We adopt specifically the formalism as summarized by Spiess [8]. The longitudinal and transverse relaxation rates of a quadrupolar nucleus, defined as the inverses of the corresponding relaxation times $T_{1}$ and $T_{2}$, are given by

(1) $$R_{1}=\frac{1}{T_{1}}=C_{Q}\;G_{2,0}\;;\;R_{2}=\frac{1}{T_{2}}=C_{Q}\;G_{2,1}\;\text{with}\;C_{Q}=\frac{e^{2}Q^{2}\left(2I+3\right)}{40I^{2}\left(2I-1\right)\hbar^{2}}$$

In the previous expressions, e is the unit charge, ℏ the reduced Planck constant, and $C_{Q}$ is a common constant dependent on the spectroscopic quadrupole moment Q and the spin I of the nucleus of interest. The quantities $G_{2,m}$ with m=−2,−1,0,1,2 (subscript “2” reflects the fact that the EFG is a rank‐2 tensor) are linear combinations of so‐called spectral densities $g_{2,m}\left(\omega \right)$, with $\omega_{0}$ being the Larmor angular frequency,

(2a) $$G_{2,0}=\;4g_{2,2}\left(2\omega_{0}\right)+g_{2,1}\left(\omega_{0}\right)+g_{2,-1}\left(-\omega_{0}\right)+4g_{2,-2}\left(-2\omega_{0}\right)$$

(2b) $$G_{2,1}=\;2g_{2,-2}\left(-2\omega_{0}\right)+3g_{2,-1}\left(-\omega_{0}\right)+2g_{2,1}\left(\omega_{0}\right)+3g_{2,0}\left(0\right)$$

The spectral densities for the quadrupolar relaxation, $g_{2,m}\left(\omega \right)$, are half‐Fourier transforms

(3) $$g_{2,m}\left(\omega_{0}\right)=\int_{0}^{\infty }f_{2,m}\left(t\right)e^{i\omega_{0}t}\mathrm{dt}$$

where $f_{2,m}$ are time‐autocorrelation functions (ACFs)
(4)

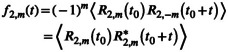




The ACFs in Equation (4) depend on the elements of the EFG in its spherical tensor representation, $R_{2,m}\left(t\right)$. The latter are defined in terms of the Cartesian elements ($u,v\in \left\{x,y,z\right\}$) of the symmetric traceless EFG tensor elements $V_{u,v}\left(t\right)$ via $R_{2,0}=3V_{\mathrm{zz}}/\sqrt{6},R_{2,\pm 1}=\mp V_{\mathrm{xz}}-{\mathrm{iV}}_{\mathrm{yz}},R_{2,\pm 2}=\left(V_{\mathrm{xx}}-V_{\mathrm{yy}}\right)/2\pm {\mathrm{iV}}_{\mathrm{xy}}$. Finally, $V_{u,v}\left(t\right)$ is calculated along the MD trajectories. It is understood that the real part of the integral on the right hand side of Equation (3) is to be used. Note, also, that the ACFs $f_{2,m}\left(t\right)$, with m=0,±1,±2, are understood to be ensemble averages of the EFG with respect to some arbitrarily chosen time origin $t_{0}$, as indicated by the notation $\left\langle \cdots \right\rangle$. Since the time steps in the MD simulations are discretized, the ACFs are likewise calculated via discrete numerical methods as detailed later.

Based on the ACFs, each EFG spherical tensor element has a characteristic *correlation time* defined as follows:

(5) $$\tau_{2,m}=\frac{1}{\sigma_{2,m}}\int_{0}^{\infty }f_{2,m}\left(t\right)\;\mathrm{dt}$$

In the denominator is $\sigma_{2,m}=f_{2,m}\left(0\right)$, which represents the ensemble variance of the corresponding EFG tensor elements in the system. For times much greater than $\tau_{2,m}$, the EFG completely loses its correlation with the initial value. Thus, $f_{2,m}$ goes to zero for $t\gg \tau_{2,m}$.

For rotationally invariant (isotropic) systems, all components of $f_{2,m}$ must be equal [57]. Furthermore, if the *extreme narrowing* (EN) condition applies, the spectral densities $g_{2,m}$ become independent of $\omega_{0}$. The EN condition implies $\omega_{0}\tau_{2,m}\ll 1$. (The validity of the EN condition may be explicitly tested by computing relaxation rates at the Larmor frequency and comparing them with zero‐frequency results, in other words, with the spectrometer magnetic induction $B_{0}$ set to 0). In practice, EN means that the range of times t in which an ACF $f_{2,m}\left(t\right)$ differs substantially from zero, that is, when it contributes to the integrand in Equation (3), corresponds to $\omega_{0}t$ being very small. Then, to a very good approximation, one can use $\omega_{0}t≃0$ for the relevant time interval over which $f_{2,m}\left(t\right)$ is non‐zero, which leads to

(6) $$g_{2,m}^{\mathrm{EN}}=\int_{0}^{\infty }f_{2,m}\left(t\right)\mathrm{dt}=\tau_{2,m}\;\sigma_{2,m}$$

As mentioned, the $g_{2,m}$ for different m become equal if the particles in the system rotate freely and rapidly on the NMR time scale. This ultimately leads to the longitudinal and transversal relaxation times becoming equal. The relaxation rate expected for an isotropic system in the EN regime is then given by

(7) $$R_{\mathrm{EN}}=\frac{1}{T_{1}^{\mathrm{EN}}}=\frac{1}{T_{2}^{\mathrm{EN}}}=10C_{Q}\;\tau_{\mathrm{iso}}\;\sigma_{\mathrm{iso}}$$

where $\tau_{\mathrm{iso}}$ and $\sigma_{\mathrm{iso}}$ correspond to the equivalent $\tau_{2,m}$ and $\sigma_{2,m}$ for an isotropic system. Note that $\sigma_{\mathrm{iso}}$ is in the quadrupolar relaxation rate literature often written as $\left\langle V{\left(0\right)}^{2}\right\rangle$.

## 3 Computational Details

Ionic liquids, particularly protic liquids, are challenging systems to describe using force fields. While force fields are essential for studying protic ionic liquids, they are often unable to model the subtle balance between long‐range Coulombic forces and short‐range hydrogen bonding. Without accounting for polarization effects, such simulations frequently fail to replicate the correct structural dynamics and ion pairing behavior inherent to these complex systems [49, 50, 58, 59]. Thus, we adopted aiMD based on the DFT electronic structure to describe the time evolution of the EAN. A trade‐off, in terms of approximations, is given by the necessity to use small simulation cells compared to those used in force field MD. The molecular dynamics simulation were performed for fully deuterated ethylammonium nitrate (EAN‐8D) to achieve a better separation of electronic and nuclear degrees of freedom [60]. Note that experimental data for the NMR relaxation, used for comparisons, are for N‐deuterated EAN (EAN‐3D) [37]. Experimental data for diffusion coefficients are for non‐deuterated EAN (EAN‐8H) [61]; a literature search did not uncover experimental diffusion coefficients of fully or partially deuterated species. The cubic simulation cell contained 15 ion pairs, totaling 225 atoms, with a cell dimension of 13.03 Å, subject to periodic boundary conditions. This simulation box, upon replacement of D with H, matched the density of undeuterated EAN, which is $1.216\;g\cdot {\mathrm{cm}}^{-3}$ at $27^{{}^{\circ}}C$ [62]. To improve the sampling and better represent isotropic conditions, the relaxation rate calculations were based on four independent MD trajectories [10]. The initial configurations were generated from a cation‐anion pair by varying their relative positions and subjecting 15 of such pairs to a packing procedure using the PACKMOL tool [63].

The aiMD simulations were carried out using the CP2K software [64]. The non‐hybrid Perdew‐Burke‐Ernzerhof (PBE) exchange‐correlation functional [65] was combined with polarized double‐zeta basis sets of VandeVondele and Hutter [66], together with the pseudopotentials proposed by Goedecker, Teter, and Hutter [67] and optimized for PBE [68]. Dispersion interactions were treated via the Grimme D3 model with Becke‐Johnson damping [69]. The aiMD simulation stage began with a geometry optimization to relax the initial‐guess cell packing, followed by 5 ps of thermalization at constant volume & temperature (NVT) at 300 K using the Nosé‐Hoover thermostat chain [70, 71]. The subsequent production phase of MD was performed in the NVE ensemble with 611 ps of duration and a time step of 0.5 fs. The first picosecond of NVE simulation was used for re‐equilibration and discarded in the relaxation rate calculations [10]. The liquid phase structure of the simulated IL was evaluated using spherical atomic radial distribution functions (RDFs) $g_{\mathrm{ij}}\left(r\right)$. RDFs were calculated for all trajectories using the VMD software [72], taking evenly spaced MD configurations (“snapshots” with 2.5 fs spacing) from the NVE production step. When, during the course of this study, it became evident that rather long aiMD simulations are needed to describe the dynamic behavior of the IL, a total of 4 independent trajectories with 611 ps duration of the production run were generated.

Self‐diffusion coefficients for the anion and cation were obtained from the ensemble‐averaged mean‐square displacement (MSD) via the Einstein relation [73]

(8) $$D=\underset{t\to \infty }{\lim}\frac{d}{\mathrm{dt}}\frac{1}{6}\left\langle {\left|r_{i}\left(t\right)-r_{i}\left(t_{0}\right)\right|}^{2}\right\rangle ,$$

where $r_{i}\left(t\right)$ is an ion's center‐of‐mass position. The self‐diffusion coefficients of the cation and anion were determined from one of the full‐length MD trajectories. The trajectory was sampled every 2.5 fs, and the MSD of the ion center of mass was obtained using the Trajectory Analyzer and Visualizer (TRAVIS) [74, 75]. To calculate the cation and anion diffusion coefficients, the respective MSD(t) functions were divided into regular time intervals. The derivative in Equation (8) was obtained from a linear regression performed for each interval. This procedure yielded several values for the diffusion coefficients, enabling us to assess the influence from the MSD time lag.

The average number of cations and anions surrounding a given ethylammonium cation was determined by analyzing the C:C and C:O RDFs [i:j notation referring to the indices in $g_{\mathrm{ij}}\left(r\right)$] resulting from the MD simulations. The protocol of extracting finite clusters from the MD for the purpose of quadrupolar relaxation calculations was extensively tested in related previous work [10]. Given that each aiMD simulation in this work contained 15 ion pairs, this led to the extraction of 15 clusters per snapshot per trajectory, containing one central cation with varying numbers of nearest neighbor (NN) species, which were used to compute EFG tensors. The NN selection was carried out using PBC3, an open‐source Fortran‐90 program developed in‐house [76]. The selection of time steps between snapshots for the purpose of calculating the EFG ACFs is discussed in some detail later.

EFG tensors at the deuterium sites of EAN‐3D were computed using the electronic structure program ORCA [77], version 6.0.1. This choice was made in part to assess DFT with different types of approximate functionals, some including exact exchange and dispersion. It is worth noting that DFT generally performs well in describing EFGs for main group atoms [11, 27, 78, 79, 80]. The EFG tensors in the present study were calculated using the range‐separated hybrid ωB97X‐V functional [81]. The “‐V” in the acronym indicates that the functional includes a dispersion term based on the VV10 correlation functional of Vydrov and Van Voorhis [82] and was parameterized accordingly. A polarized valence triple‐zeta basis minimally augmented with diffuse functions, ma‐def2‐TZVP [83], was used for all atoms, because the IL contains negatively charged ions. Additional functionals and basis sets were tested for the EFG calculations, as detailed in the Results and Discussion section.

Bulk effects from the IL were added to the cluster models via the universal solvation model (SMD) [84]. It has been shown that SMD can describe free solvation energies of neutral solutes in ionic liquids, with errors similar to those typically observed for SMD in ordinary liquids [85]. Within SMD, the primary solvent descriptors are the dielectric constant, refractive index, macroscopic surface tension, and Abraham‐type acidity and basicity parameters. Those properties for EAN are available in the literature [86, 87, 88], except for the acidity and basicity parameters. For the latter, we used those of the “generic ionic liquid” defined in Reference [85].

As mentioned, we developed a new open‐source Fortran (F90) code ‘QRELAX’ to generate quadrupolar relaxation rates based on the raw data generated from the MD configurations [89]. The approach is summarized as follows: From a set of evenly time‐spaced MD configurations, the EFG tensors are calculated as described in the previous paragraphs. The EFG ACFs are then evaluated based on the Wiener‐Khinchin Theorem, which states that the time‐ACF of a function $R\left(t\right)$ with Fourier transform $r\left(\omega \right)$ is equal to the (inverse) Fourier transform of ${\left|r\left(\omega \right)\right|}^{2}$. For this, QRELAX implements a discrete Fourier transform according to Reference [90]. The half‐Fourier transform of $f_{2,m}\left(t\right)$ is obtained by numerical integration using Simpson's rule and cubic spline interpolation according to Reference [90]. The same integration scheme was used to calculate the correlation times $\tau_{2,m}$. QRELAX includes data sets of gyromagnetic ratios and Q values taken from the open‐source EasySpin software [91] and Pyykkö's most recent collection of nuclear quadrupole moments [92], respectively. The QRELAX input file requires only minimal further information, such as the Larmor frequency of the proton used to establish the value of $B_{0}$, and the isotope of interest. The correctness of QRELAX results was assessed by direct comparison with DynPy [93], a Python‐based code for calculating NMR relaxation rates and other dynamic properties from MD simulations developed previously in one of our laboratories [10, 17, 27, 94]. The development of QRELAX was deemed necessary for a variety of reasons which include code maintainability and numerical performance. Finally, since ethylammonium has three NMR‐equivalent deuterium nuclei attached to the nitrogen, the corresponding autocorrelation functions were averaged, resulting in a single $f_{2,m}$ function per cation used to compute the spectral density $g_{2,m}^{Q}$. The data for different cations (and from the different trajectories, except for some of the test calculations) were averaged subsequently. Relaxation rates under the EN condition were computed by setting $B_{0}=0$ in the input of QRELAX and replacing each $f_{2,m}$ with an average over the five different m (option ForceIsotropy).

## 4 Results and Discussion

The adopted methodology requires one or more MD simulations of the system to generate a set of configurations evenly spaced in time, which are used to determine the EFG tensor and the associated ACFs. The main aspects related to the MD simulations are discussed first.

### 4.1 Ab‐Initio Molecular Dynamics

Radial pair‐RDFs were constructed for all MD trajectories to assess the liquid structure of the IL. In a neutron diffraction‐based study, Hayes et al. determined 13 different RDFs from diffraction patterns of EAN with different isotopic substitutions [95], using the Empirical Potential Structure Refinement (EPSR) [96] model for a Monte Carlo simulation. Further details are specified in the cited literature. The EPSR model also includes an empirical potential that is iteratively refined, such that the simulated diffraction patterns fit the experimental data. We compare our simulation results with those based on the experiment‐guided EPSR, referred to as “experimental” in what follows. Table 1 presents the positions of the first two peaks of RDFs (averaged over the four aiMD simulations) involving pairs of atoms that are relevant to characterize the neighborhood around the cations and anions. Plots of the individual and averaged RDFs are available in the Figures S1 and S2.

**FIGURE 1:**
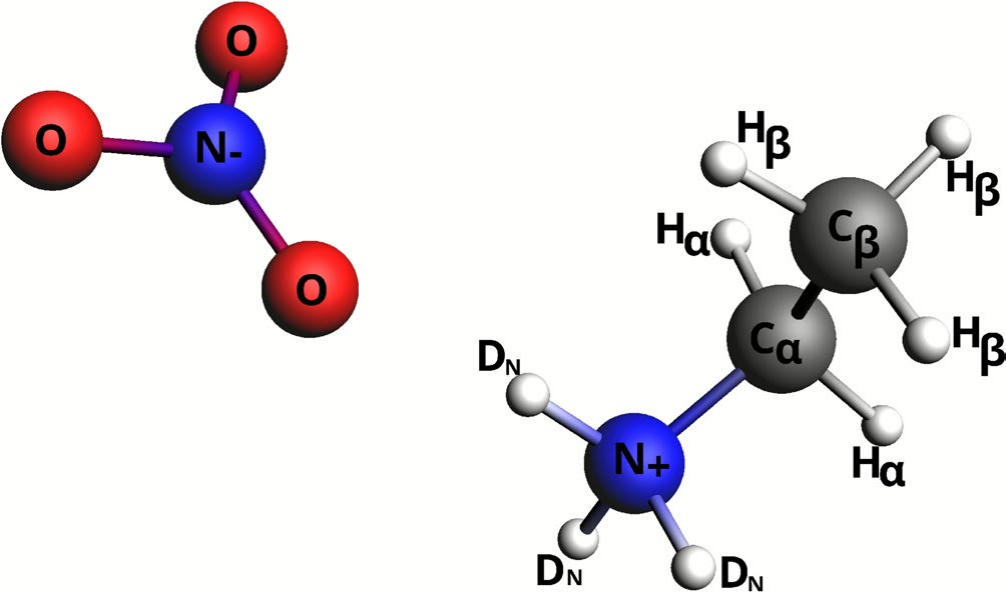
Atom labels used in Table 1. The image was generated from a snapshot from one of the MD simulations.

**TABLE 1:** Peak positions (in Å) in the radial pair‐distribution functions for EAN. See Figure 1 regarding the atom labels.^a^

Ion pair	Atom pair	First shell		Second shell	
		aiMD	EPSR	aiMD	EPSR
Anion: anion	$N_{-}:N_{-}$	≈4	3.3	5.45	5.9
	$N_{-}:O$	4.55	3.0	5.65	4.6
Cation:cation	$C_{\beta }:C_{\beta }$	3.90	3.5	7.50	—
	$C_{\beta }:C_{\alpha }$	4.05	3.9	5.05	4.7
	$C_{\beta }:N_{+}$	5.25	5.4	—	—
	$C_{\alpha }:C_{\alpha }$	5.25	5.0	—	—
	$N_{+}:N_{+}$	5.05	5.4	—	—
	$H_{\beta }:H_{\beta }$	≈3	2.6	4.05	3.6
Cation:anion	$C_{\beta }:N_{-}$	4.20	4.1	7.95	—
	$N_{+}:O$	2.85	3.0	4.65	4.7
	$N_{-}:N_{+}$	3.45	3.6	8.45	—
	$N_{-}:C_{\beta }$	4.20	4.3	7.95	—
	$D_{N}:O$	1.75	2.4	3.25	3.9

*Note:* Results from aiMD simulations for EAN‐8D (present work) vs. EPSR data from Reference [95].

^a^ ${\text{N}}_{-}$: anion nitrogen; $N_{+}$: cation nitrogen; $C_{\alpha }$ and $C_{\beta }$: α and β‐carbons to the amine group; $H_{\beta }$: hydrogen attached to a β‐carbon; $D_{N}$: deuterium attached to a nitrogen.

Considering the structure related to anions surrounding an anion, the first peak of the RDF involving pairs of nitrogen appears mostly as a “shoulder” around 4 Å. The experimental value is 3.3 Å. The first peak of the nitrogen‐oxygen RDF occurs at 4.55 and 3.0 Å, respectively, for aiMD and EPSR. Therefore, the aiMD gives a less tightly packed nitrate layer compared to the EPSR. When assessing the distances involving pairs of atoms belonging to cations, we find that the aiMD values agree reasonably well with those derived via EPSR. Likewise, the RDF peaks involving cation‐anion pairs agree well between aiMD and EPSR, except for the peak locations from the $g_{D_{N},O}\left(r\right)$ RDF, which are somewhat smaller than the experimental reference values. However, it is worth mentioning that very similar results were observed in other aiMD studies based on GGA functionals [54, 97], while at the same time it needs to be kept in mind that the “experimental” RDF peak positions are based on a procedure that involves a considerable amount of theoretical modeling as well. Therefore, while it may be the case that the present aiMD simulations afford a degree of over‐structuring of the IL, perhaps similar to what has been reported previously for liquid water [60], the liquid structure of EAN appears to be reasonably well described by the aiMD simulations.

**FIGURE 2:**
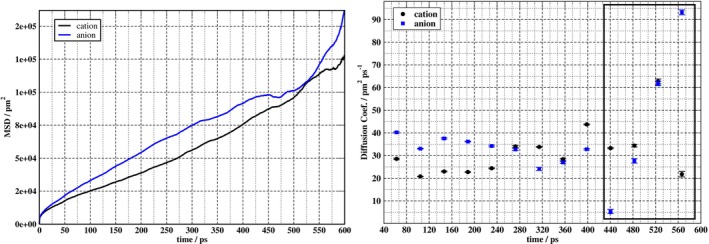
NEA‐8D cation and anion mean‐square displacements MSD (t) (left panel). Diffusion coefficients (right panel), calculated from MSD values taken at regular intervals of 40 ps.

In addition to perusing the RDFs, the reliability of MD may be assessed by means of self‐diffusion coefficients. As mentioned, the diffusion coefficients of the cation, $D_{+}$, and the anion, $D_{-}$, were calculated using the Einstein relation (Equation (8)). This approach potentially introduces a certain arbitrariness in the results because of the procedure adopted to calculate the derivative of the MSD over long times. The derivative is usually obtained from the slope of a linear regression of the MSD as a function of time. However, only an intermediate segment of time should be used for the MSD calculation [98] because, initially, the system is in the so‐called ballistic regime, while toward the end of the sampled time interval the MSD is subject to errors from lack of sampling. To choose the most suitable time interval for MSD $\left(t\right)$ in Equation (8), 1000 values of the function were computed for the cation (anion), comprising the interval 0−600 ps (Figure 2), which was subdivided into 14 regular intervals of approximately 40 ps. The diffusion coefficients obtained for each interval are shown in Figure 2, with the data point time values corresponding to the center of each time interval. Per the data in Figure 2, we note that for t<240 ps, the anion coefficient is larger than that of the cation, which is in agreement with the experiment. The ordering of the diffusion coefficients switches in the interval 270−400 ps and is followed by a buildup of significant scatter in $D_{+/-}$ for t>400 ps, which is attributed to insufficient sampling. It would appear to be necessary to extend the simulation time much beyond 600 ps so that the MSD function can be adequately estimated up to 600 ps, thus fulfilling the limiting condition of Equation (8). For this reason, we selected the diffusion coefficients obtained at 230 ps for the diffusion coefficients for assessing the aiMD.

Accordingly, the estimated values for the diffusion coefficients of the EAN ions in the aiMD are $D_{+}=\left(24.43\pm 0.16\right)$ and $D_{-}=\left(34.22\pm 0.35\right)$ in units of $10^{-12}\;m^{2}\;s^{-1}$. At 300 K, the experimentally determined values for these coefficients (for non‐deuterated EAN, as mentioned) are $D_{+}=\left(46.20\pm 0.02\right)$ and $D_{-}=\left(78.0\pm 0.7\right)$ in the same units [61]. The aiMD cation and anion coefficients underestimate the values extracted from measurements, but they are of the right order of magnitude. Underestimated diffusion coefficients may potentially reflect the tendency of GGA functionals to over‐structure the hydrogen bonding network, which was previously been noted for liquid water [60]. Since hydrogen bonds are also present in PILs, the ion mobility may be reduced. However, it is at present unclear if the known shortcomings for aiMD simulations of pure water with GGA functionals extend to other hydrogen‐bonded systems as well. Another potential source of error is the finite size of the simulation cell as used in this study with periodic boundary conditions. It has been noted in this context that diffusion coefficients obtained through molecular dynamics simulations with periodic boundary conditions exhibit a non‐negligible dependence on the system size [99, 100, 101]. Overall, however, the diffusion coefficients and RDF peaks obtained from the aiMD appear to be in reasonable agreement with available experimentally derived data, such that we consider an equivalent setup for EAN‐3D suitable for the targeted study of NMR relaxation.

### 4.2 Quadrupolar Relaxation

Determining the longitudinal ($R_{1}=1/T_{1}$) and transverse ($R_{2}=1/T_{2}$) relaxation rates requires knowledge of the temporal evolution of the EFG at the quadrupolar nucleus site. This sub‐section considers relevant aspects related to the EFG calculation and sampling.

#### 4.2.1 Clustering

The chosen computational protocol rests on aiMD with periodic boundary conditions coupled with calculations of the EFG tensors for finite clusters augmented with a solvent model for bulk effects. In the context of the present work, this concerns the calculation of the EFG tensors of the deuterium atoms in the IL cationic component. In terms of sampling, it is advantageous to consider all cations (15, in the present study) in the simulation box. Consequently, we adopted the following procedure for calculating the EFGs: (i) Configurations are extracted from the molecular dynamics trajectory at regular time intervals according to the preceding discussion. (ii) Each configuration under periodic boundary conditions is converted into a finite 3 × 3 × 3 supercell. (iii) EAN‐3D cations from the original simulation cell and a select number of NN ions (extending into neighboring cells, when needed) are selected as described in the Computational Details. The question then becomes how many NN ions need to be extracted such that the relaxation properties in the calculation are converged.

**FIGURE 3:**
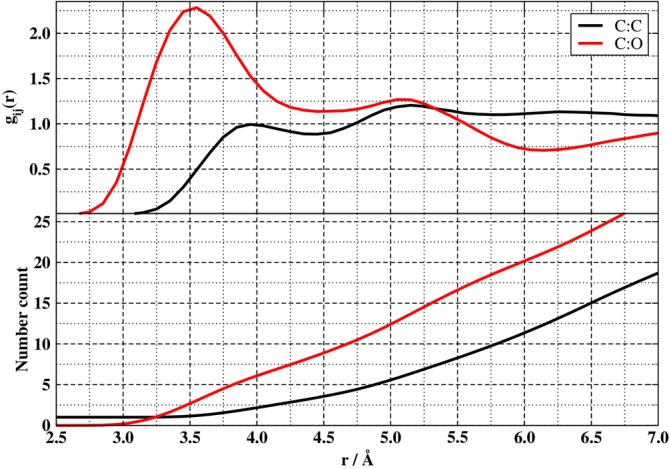
Radial distribution functions $g_{\mathrm{ij}}\left(r\right)$ for atom pairs i:j as indicated in the legend, obtained from trajectory one. The reference atom i is any carbon in ethylammonium. Atom j = C is the other carbon in the same or one of the carbons in another ethylammonium cation. Atom j = O refers to any of the nitrate oxygens. The panel below the RDFs, showing the number count, was generated by integrating those functions.

Figure 3 displays selected RDFs and their radial integrations for trajectory one, where $g_{\mathrm{CC}}\left(r\right)$ (black curves) refers to any of the carbons in the cation, while the atom pair in the $g_{\mathrm{CO}}\left(r\right)$ RDF (red curves) involves any carbon of the ethylammonium and an oxygen of the nitrate. It can be deduced from the RDFs in Figure 3 that a first shell of cations and anions covers a radius from the reference atom of about 4.50 Å and encompasses on average up to two cations and three anions. These findings are in agreement with experimental data [95]. Within a distance of about 6.25 Å, the cation is surrounded by its first and second shells, containing in aggregate about six cations and seven anions. The PBC3 program used to extract the clusters from the MD configurations will extract a user‐specified number of NN. However, the number of cations versus anions among those NN may vary from configuration to configuration depending on the progress of the dynamics. For example, during the MD another cation might move closer to the analyte cation while at the same time one of the anions moves further away. This might then lead to a different composition of the analyte‐NN cluster in terms of the number of cations vs. anions. However, the presence of the SMD solvent model in the finite‐cluster calculations will render implicit representations of the more distant species in case they move out of the cluster that is treated quantum mechanically.

To determine the number of neighbors appropriate to represent the explicit solvation of the cations, relaxation properties were calculated considering 5 and 13 nearest neighbors, corresponding to the first and first two shells, respectively, surrounding the cation in the IL. According to the data in Table 2, the addition of the second shell around the cations in the EFG calculations only has a very small effect on their relaxation properties, not exceeding 4%. The relative signed differences between the data for 5 and 13 NN become even smaller when the data for the individual cations are averaged. This result is in line with the fact that quadrupolar relaxation is driven by the EFG, which itself is strongly dominated by local contributions, such that if the solvation spheres around the nucleus whose relaxation is being observed are representative of the real liquid, the autocorrelation functions are expected to be adequately described. The situation is quite different when considering relaxation via the dipolar mechanism, which requires a correct description of the motion of a pair of spins interacting over rather large distances. Indeed, in References [101, 102], for example, concerning the relaxation of protons, considerable portions of these works are directed at the impact of the box size, PBC, and duration of the production step on the corresponding autocorrelation functions and spectral densities.

**TABLE 2:** Deuterium relaxation parameters calculated for individual cations of EAN‐3D surrounded by 5 vs. 13 nearest neighbors (NN).^a^

Cation	NN	$R_{1}$		$R_{2}$		$R_{\mathrm{EN}}$		$\sigma_{\mathrm{iso}}$		$\tau_{\mathrm{iso}}$	
1	5	7.279		9.335		9.041		2.349		17.295	
	13	7.238	(0.6)	9.463	(−1.3)	9.108	(−0.7)	2.365	(−0.6)	17.311	(−0.1)
2	5	13.755		7.682		10.402		2.424		19.287	
	13	13.823	(−0.5)	7.767	(−1.1)	10.499	(−0.9)	2.440	(−0.6)	19.341	(−0.3)
3	5	7.965		6.443		8.329		2.337		16.020	
	13	7.886	(1.0)	6.458	(−0.2)	8.336	(−0.1)	2.354	(−0.7)	15.914	(0.7)
4	5	8.884		8.855		8.860		2.290		17.389	
	13	9.000	(−1.3)	8.810	(0.5)	8.875	(−0.2)	2.304	(−0.6)	17.310	(0.5)
5	5	16.903		17.463		16.300		2.325		31.512	
	13	17.243	(−2.0)	17.617	(−0.9)	16.494	(−1.2)	2.342	(−0.7)	31.650	(−0.4)
6	5	14.081		16.069		15.743		2.400		29.480	
	13	14.187	(−0.7)	16.218	(−0.9)	15.863	(−0.8)	2.420	(−0.8)	29.454	(0.1)
7	5	7.329		8.660		7.193		2.424		13.337	
	13	7.598	(−3.5)	9.013	(−3.9)	7.482	(−3.9)	2.447	(−0.9)	13.743	(−2.9)
8	5	21.097		26.494		24.796		2.290		48.654	
	13	21.056	(0.2)	26.646	(−0.6)	24.874	(−0.3)	2.302	(−0.5)	48.557	(0.2)
9	5	4.974		6.379		5.872		2.354		11.213	
	13	5.044	(−1.4)	6.413	(−0.5)	5.924	(−0.9)	2.371	(−0.7)	11.228	(−0.1)
10	5	8.203		17.610		12.991		2.324		25.126	
	13	8.238	(−0.4)	17.736	(−0.7)	13.071	(−0.6)	2.342	(−0.8)	25.080	(0.2)
11	5	8.461		9.493		9.449		2.367		17.943	
	13	8.490	(−0.3)	9.526	(−0.3)	9.500	(−0.5)	2.390	(−1.0)	17.864	(0.4)
12	5	8.559		4.687		5.773		2.379		10.904	
	13	8.666	(−1.2)	4.671	(0.4)	5.805	(−0.6)	2.399	(−0.8)	10.873	(0.3)
13	5	5.571		6.193		6.411		2.355		12.233	
	13	5.655	(−1.5)	6.205	(−0.2)	6.454	(−0.7)	2.372	(−0.7)	12.229	(0.0)
14	5	19.506		16.909		16.149		2.313		31.372	
	13	19.527	(−0.1)	16.745	(1.0)	16.034	(0.7)	2.320	(−0.3)	31.064	(1.0)
15	5	8.000		6.980		6.975		2.381		13.166	
	13	8.078	(−1.0)	7.009	(−0.4)	7.011	(−0.5)	2.398	(−0.7)	13.142	(0.2)
Mean	5	10.705		11.283		10.952		2.354		20.909	
	13	10.782	(−0.7)	11.353	(−0.6)	11.022	(−0.6)	2.371	(−0.7)	20.891	(0.1)

^a^ EFGs computed at the ωB97X‐V/SCNL/ma‐def2‐TZVP level at time intervals of 0.25 ps. $R_{1}$, $R_{2}$, and $R_{\mathrm{EN}}$ are relaxation rates (Hz), $\sigma_{\mathrm{iso}}$ is the EFG variance (×100, in a.u.), and $\tau_{\mathrm{iso}}$ the correlation time (ps). Data for the relaxation rates and associated properties were collected from MD trajectory no. one. Percent differences between the results obtained with 13 NN compared to 5 NN are given in parentheses.

Since EAN has a molecular cation, it is conceivable that the most significant contributions to the ACFs and therefore the relaxation rates are caused by the cation's internal motion, which would be adequately sampled by the MD and the resulting clusters even with a small number of NN. At this point, it would be valuable to assess the contribution of the bare cation versus its solvated form to evaluate the importance of explicit solvation of the cations. We performed a calculation of the $R_{1}^{\mathrm{iso}}$ rate (ForceIsotropy option) for one of the cations along trajectory no. 1, without the presence of any neighboring ions in the EFG calculations, using only implicit SMD solvation. The rate obtained for the isolated cation is approximately 30% higher than that of the 13 NN solvated form. Both the variance of the spherical components of the EFG and the correlation time are affected by the elimination of neighboring ions. They each increased by more than 10%. Thus, internal motions have a significant contribution to the ACFs, but explicit solvation is also important. Therefore, we conclude that the inclusion of the first two NN shells of the cations in EAN‐3D adequately represents the specific interactions relevant to the deuterium quadrupolar relaxation. This setup was therefore adopted for generating the results discussed in the remainder of this article.

#### 4.2.2 XC‐Functional and Basis Set Dependency

To evaluate the effect of the level of theory used for the EFG calculations on the EAN‐3D relaxation properties, different exchange‐correlation functionals and basis sets were combined. This assessment used one of the 15 cations of trajectory one, to save computational resources. Selected functionals were the global hybrid PBE0 [103] and the range‐separated hybrid GGA ωB97X‐V and hybrid meta‐GGA ωB97M‐V [104]; the last two include a non‐local dispersion term (VV10) [82] as explained in the Computational Details. For the latter, and for added VV10 corrections in PBE0, we adopt the ORCA manual notation where self‐consistent KS calculations including VV10 are indicated by the ‘SCNL’ acronym (Self‐Consistent computations with Non‐Local density‐dependent dispersion correction). The EFG tensors were calculated with the aforementioned functionals and the basis sets def2‐TZVP [105], ma‐def2‐TZVP [83], and def2‐VZVPD [106]. The computed properties are the longitudinal and transverse relaxation rates of the system in an external magnetic field of 11.7 T, the rate $R_{\mathrm{EN}}$ under the extreme narrowing condition, the variance of the EFG ($\sigma_{\mathrm{iso}}$), and the correlation time defined via Equation (7).

The PBE0 results, gathered in Table 3, indicate that these properties are only slightly sensitive to the addition of diffuse basis functions, with differences not greater than 0.2%. The SCNL correction changes all but the $\tau_{\mathrm{iso}}$ results by about 1%. The correlation time changes by less than 0.1%. When PBE0 is replaced with ωB97X‐V, relaxation rates and EFG variance increase by 2%, but again, $\tau_{\mathrm{iso}}$ is practically unchanged. The difference between ωB97X‐V and ωB97M‐V is small, not more than 0.2%. Also, the differences between the two basis sets containing diffuse functions are less than 0.8%, with ma‐def2‐TZVP being more compact. Therefore, we adopted the ωB97X‐V/SCNL/ma‐def2‐TZVP level for all subsequent EFG calculations.

**TABLE 3:** Deuterium relaxation data for a single cation cluster of EAN‐3D, depending on the DFT functional and basis set chosen for the EFG calculations.^a^

	$R_{1}$	$R_{2}$	$R_{\mathrm{EN}}$	$\sigma_{\mathrm{iso}}$	$\tau_{\mathrm{iso}}$
PBE0/def2‐TZVP	13.489	7.586	10.250	2.385	19.311
PBE0/ma‐def2‐TZVP	13.517	7.602	10.272	2.390	19.314
PBE0/SCNL/def2‐TZVP	13.634	7.666	10.359	2.409	19.324
PBE0/SCNL/ma‐def2‐TZVP	13.662	7.683	10.381	2.414	19.328
ωB97X‐V/SCNL/ma‐def2‐TZVP	13.823	7.767	10.499	2.440	19.341
ωB97X‐V/SCNL/def2‐TZVPD	13.737	7.713	10.429	2.428	19.305
ωB97M‐V/SCNL/ma‐def2‐TZVP	13.841	7.780	10.514	2.437	19.386

^a^ Data for the relaxation rates and associated properties were collected from MD trajectory no. one. Relaxation rates $R_{1}$, $R_{2}$, and $R_{\mathrm{EN}}$ in Hz, EFG variance $\sigma_{\mathrm{iso}}$ times 100 in a.u., correlation time $\tau_{\mathrm{iso}}$ in pico‐seconds.

#### 4.2.3 Configuration Sampling Time Steps

Apart from technical details related to the MD, the relaxation rate calculation itself does not require much input. Some of the input is related to the experimental conditions, e.g., the Larmor frequency of the relevant nuclide. An additional, critical input is the temporal sampling frequency used to compute the ACFs, which is controlled via the input variable TimeStep. The temporal EFG sampling rate and the total time sampled control the convergence of the Fourier transform used to obtain the ACF via the Wiener‐Khinchin theorem. To assess the sensitivity of the relaxation properties to this parameter, data were collected from a single cation cluster of trajectory one (the same as in the previous section), with EFG sampling time steps varying from 1 to 0.0625 ps. The resulting relaxation data are collected in Table 4.

**TABLE 4:** Deuterium relaxation parameters for a single cation cluster of EAN‐3D as a function of the EFG sampling time step (ps).^a^

Time step	$R_{1}$		$R_{2}$		$R_{\mathrm{EN}}$		$\sigma_{\mathrm{iso}}$		$\tau_{\mathrm{iso}}$	
1.0000	13.595	(−1.0)	7.887	(−0.5)	10.423	(−1.1)	2.409	(−1.1)	19.445	(−0.0)
0.5000	13.608	(−0.9)	7.779	(−1.9)	10.389	(−1.4)	2.421	(−0.6)	19.282	(−0.8)
0.2500	13.823	(0.7)	7.767	(−2.1)	10.499	(−0.4)	2.440	(0.2)	19.341	(−0.5)
0.1250	13.619	(−0.8)	7.813	(−1.5)	10.432	(−1.0)	2.439	(0.1)	19.225	(−1.1)
0.0625	13.729		7.930		10.541		2.436		19.447	

^a^ Numbers in parentheses are percent differences relative to the 0.0625 ps time step data. EFGs computed with ωB97X‐V/SCNL/ma‐def2‐TZVP. Data for the relaxation rates and associated properties were collected from MD trajectory no. one. Relaxation rates $R_{1}$, $R_{2}$, and $R_{\mathrm{EN}}$ in Hz, EFG variance $\sigma_{\mathrm{iso}}$ times 100 in a.u., correlation time $\tau_{\mathrm{iso}}$ in pico‐seconds.

The data for the shortest EFG sampling time step, 0.0625 ps, are used for reference. In comparison, it is evident that the relaxation rate data are not very sensitive to the sampling time step—as long as the latter is chosen within reasonable limits—with unsigned deviations not exceeding 2%. The apparently ‘better’ performance of the calculations with the largest EFG sampling time step (1.0000 ps) for the $R_{\mathrm{EN}}$ relaxation rate, compared to the intermediate size steps (0.5000–0.1250 ps) must be fortuitous. Obviously, the numerical representations of the ACFs should be not too coarse over a time window that is comparable to the characteristic relaxation times $\tau_{2,m}$ and $\tau_{\mathrm{iso}}$. Therefore, a sampling time step of 0.2500 ps would seem to be a good compromise between the convergence of relaxation properties and the overall computational cost and was chosen for the production calculations. As a reminder, the EFG sampling time step differs from the time step in the aiMD simulations, which was 0.5 fs.

#### 4.2.4 Independent Trajectories

**FIGURE 4:**
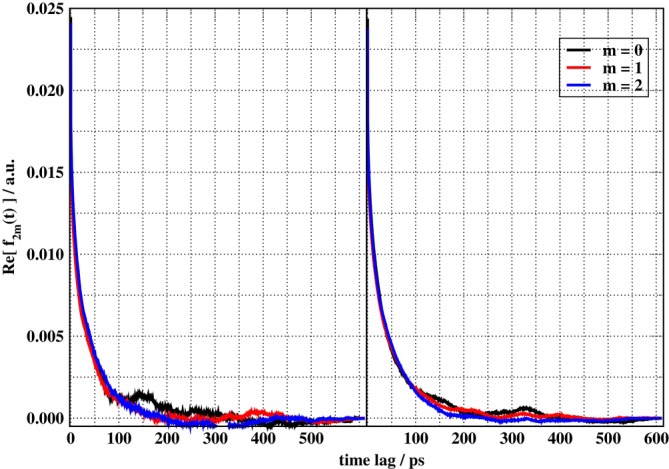
N‐bound deuterium EFG autocorrelation functions for EAN for (left) a single trajectory (no. one) and (right) averaged over four independent trajectories.

In isotropic liquids, as is the case of EAN, the isotropy condition manifests itself also in the ACFs of the EFG. This results in the ACF for each component m of the EFG being equivalent. This behavior can be illustrated graphically by plotting $f_{2,m}$ for different m. The left panel of Figure 4 presents the real parts of $f_{2,m}\left(t\right)$ for m=0,1,2, as a function of the time lag for one of the MD trajectories (no. one). The average of $f_{2,m}$ over the four EAN‐3D trajectories is shown in the right panel. The components where m is negative were omitted because $f_{2,m}=f_{2,-m}^{*}$ and therefore the real parts are the same. As can be seen, the ACFs are similar already for this one trajectory, but $f_{2,0}$ displays a much slower decay than the other two functions, becoming equal to zero around 320 ps into the dynamics. In contrast, the other two cross the zero line at about 180 ps. The right panel of Figure 4 shows the same ACFs but now averaged over the four independent trajectories. The improvement in terms of the data reflecting an isotropic IL is noticeable. Furthermore, averaging over different trajectories produces the same effect as a more extended sampling, and it also reduces some of the variations at short time lags that can be seen in the left panel of Figure 4.

Working with several independent trajectories instead of a single, more extended one may also be advantageous for better sampling the configuration space in the MD, as was pointed out already in Reference [10]. The properties determined for the different trajectories can then also be used to calculate mean values and standard deviations. This does not give access to the true sampling error, but it does provide an impression of what this error would be like if the set of trajectories were representative of the configuration space sampled by the system. In the present study of quadrupolar relaxation, there are two ways to obtain averaged quantities, either by calculating the relaxation properties for each MD trajectory and then averaging the results, or by calculating trajectory‐averaged ACFs and from those the properties. The relaxation rates are identical in both cases, as is the variance of the EFG, but the correlation times are different because of the definition in Equation (5). We chose the first option because it allows the mean and standard deviation to be easily estimated. Since each individual trajectory also produces data for the 15 cations contained in the simulation box separately, we can likewise obtain standard errors for the individual trajectories from the scatter of the data for the different cations. The relaxation data for the independent trajectories are presented in Table 5. Each row in that table represents an average over the quadrupole relaxation properties of the 15 cations in the box. The “Avg” row represents an average value computed from the properties of 60 cations from the four trajectories.

**TABLE 5:** Mean deuterium relaxation data and standard errors for EAN‐3D for the independent MD trajectories.^a^

No.	$R_{1}$	$R_{2}$	$R_{\mathrm{EN}}$	$\sigma_{\mathrm{iso}}$	$\tau_{\mathrm{iso}}$
1	10.8 ± 1.3	11.4 ± 1.6	11.0 ± 1.4	2.371 ± 0.012	21.0 ± 2.7
2	16.1 ± 2.9	17.4 ± 3.6	17.1 ± 3.1	2.352 ± 0.011	32.9 ± 6.3
3	13.5 ± 2.4	16.3 ± 2.9	15.5 ± 2.5	2.367 ± 0.011	29.7 ± 4.9
4	12.7 ± 1.2	13.6 ± 2.1	13.0 ± 1.6	2.363 ± 0.009	24.9 ± 3.2
Avg.	13.3 ± 1.0	14.7 ± 1.3	14.2 ± 1.1	2.363 ± 0.005	27.1 ± 2.3

^a^ EFGs computed with ωB97X‐V/SCNL/ma‐def2‐TZVP. $R_{1}$, $R_{2}$, and $R_{\mathrm{EN}}$ are relaxation rates (Hz), $\sigma_{\mathrm{iso}}$ is the EFG variance (×100, in a.u.), and $\tau_{\mathrm{iso}}$ the correlation time (ps).

As indicated in Table 5, the longitudinal and transverse rates vary substantially between different trajectories; for instance, $R_{1}$ varies from 10.8 to 16.1 Hz, demonstrating the importance of using multiple independent trajectories. Considering the average values $R_{1}=13.3\pm 1.0$ and $R_{2}=14.7\pm 1.3$ Hz, first to note is that they agree within the estimated standard errors. The main reason for their numerical difference is a residual imperfect description of the isotropy of the medium, even when considering four independent trajectories longer than 600 ps each. When the rates are calculated using the ForceIsotropy option, where the program replaces the ACF components by the average value, the rates $R_{1}$ and $R_{2}$ by design become equal (under the condition $\omega_{0}\tau_{2,m}\ll 1$) and adopt a value of 14.2±1.1 Hz. The experimentally determined value for $R_{1}$ is 8.03 Hz [37], which is below our result but reasonably close. For the aiMD‐ and DFT‐based methodology adopted in the present study, a ratio between calculated and measured relaxation rates of up to a factor of 3 has in the past been deemed entirely satisfactory for much simpler systems [10, 15, 16, 17, 22, 27]. We consider the limitations of GGA‐functional based aiMD in sampling the configuration space and accurately describing a system's dynamic behavior as the main limitation of the approach. Regarding the EFG correlation time, the average value obtained from the simulations is 27.1±2.3 ps. This is somewhat higher than the reference value of 16 ps, derived by combining DFT calculations and NMR measurements [37], but still quite satisfactory.

**FIGURE 5:**
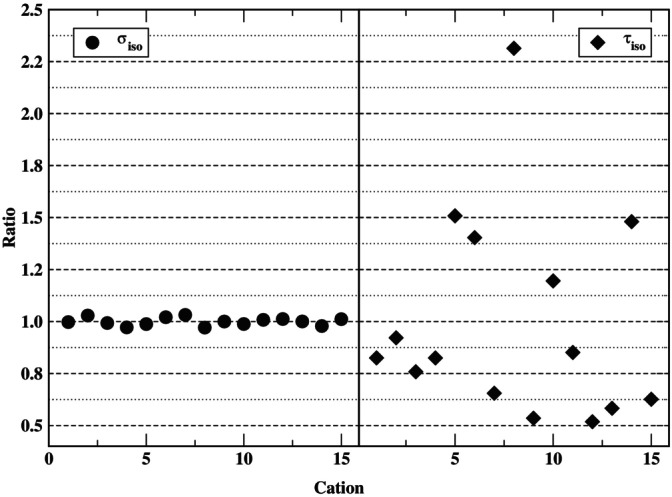
EFG variance $\sigma_{\mathrm{iso}}$ and correlation time $\tau_{\mathrm{iso}}$ for each cation of trajectory one of EAN‐3D. The vertical axes show the relevant quantity for an individual cation divided by the average.

The EFG variance $\sigma_{\mathrm{iso}}$ displays, as usual [27], the smallest relative standard deviation because it is averaged over the molecules in the simulation cell as well as time. The relaxation rates and correlation time afford larger uncertainties, but those are consistently less than 10% for the average of all trajectories. The qualitative difference in the variations of $\sigma_{\mathrm{iso}}$ vs. $\tau_{\mathrm{iso}}$, for example, can also be compared visually, as shown in Figure 5, which highlights the scatter in the latter vs. the consistency in the former. Two extreme cases are furnished by cations two and eight. They have $\tau_{\text{iso}}$ with the lowest and highest deviations from the average value (i.e., from a ratio equal to one), respectively. For both cations, the isotropic ACF (Figure S5) shows a rapid initial decay within less than 1 ps. For cation eight, rapid decay changes to a slow‐decay regime, where correlations are observed even for times close to 250 ps. Cation two, on the other hand, has a normalized ACF that decays rapidly to zero within about 130 ps and afterwards oscillates closely around zero.

#### 4.2.5 Trajectory Length

**FIGURE 6:**
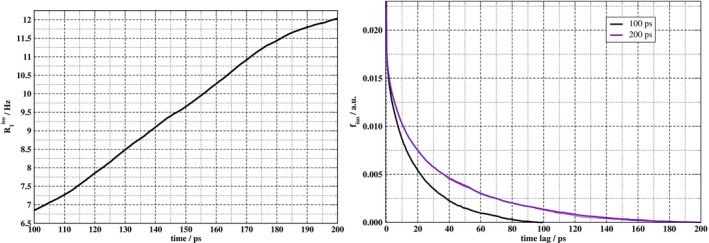
(Left) Deuterium longitudinal relaxation rate of EAN‐3D with respect to the duration of the molecular dynamics. (Right) Isotropic autocorrelation functions computed over two MD segments of different lengths of trajectory one.

Another fundamental aspect in the simulation of nuclear spin relaxation concerns the length of the molecular dynamics and the corresponding EFG autocorrelation functions. The algorithms used to calculate the ACFs numerically produce functions that decay to zero when approaching the end of the considered time window, no matter whether the ‘true’ ACF has decayed or not. Only via sampling longer time intervals can it be ascertained that the ACF has truly decayed to negligible values; otherwise, unphysically small relaxation rates may be generated. The case is illustrated by Figure 6, which shows the behavior of $R_{1}^{\mathrm{iso}}$ (averaging 15 cations and 4 trajectories) as the time window for the aiMD production run is varied between 100 to 200 ps. The relevant ACFs for 100 and 200 ps simulations are shown in the right panel of the figure. In both cases, they decay to zero within the selected time interval. However, it is clear that the decay is artificial in both cases, and the plot of the rate in the left panel for varying simulation length does not indicate convergence. Instead, the relaxation rate increases as the duration of molecular dynamics increases. This is a clear sign that 200 ps of aiMD simulation is not sufficient to converge the quadrupolar 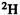 NMR relaxation rate of EAN‐3D.

**FIGURE 7:**
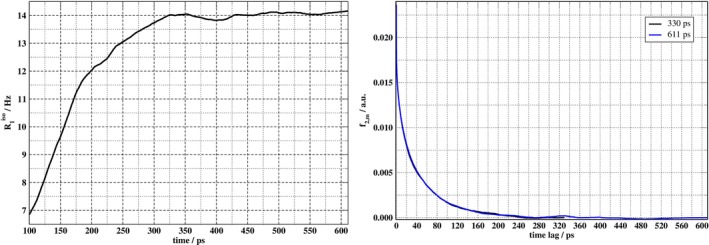
(Left) Convergence of the EAN‐3D longitudinal relaxation rate as a function of trajectory duration. (Right) Isotropic EFG autocorrelation functions for trajectories with 330 vs. 611 ps duration. Relaxation rates were calculated using an isotropic average over the components of the EFG autocorrelation functions (ForceIsotropy option).

In fact, data such as displayed in Figure 6 that we examined during the course of this study prompted what might appear to some readers as rather excessively long aiMD simulations of more than 600 ps. Namely, as illustrated in Figure 7, convergence of the relaxation rate is achieved only after more than 300 ps, with $R_{1}^{\mathrm{iso}}$ having a value of approximately 14 Hz. Note that this value for $R_{1}^{\mathrm{iso}}$ is close to the average $R_{\mathrm{EN}}$ in Table 5, indicating that the inequality $\omega_{0}\tau_{2,m}\ll 1$ applies to the quadrupolar relaxation of EAN‐3D. Small oscillations (less than 0.2 Hz) can be noted in the $R_{1}^{\mathrm{iso}}$ values within the range of 325 to 611 ps, indicating that the EFG components are uncorrelated in this time range. The autocorrelation functions for trajectories with durations of 330 versus 611 ps are compared in that figure and seen to be virtually indistinguishable on the scale of the plot, thus showing that times greater than 330 ps are sufficient for the ACF convergence (barring rare events that are not sampled but would strongly impact the ACFs). This production time is much longer than what was needed for non‐IL systems such as liquid water, benzene, or acetonitrile [17]. The need for very long simulations, by aiMD standards, is in part a consequence of the low mobility of the ions in the IL. To determine a final value of the calculated relaxation rate of deuterium in EAN‐3D, we took an average of the rates obtained with simulation lengths in the range from 330 to 611 ps. This resulted in a rate of 14.024±0.004 Hz. We reiterate that the standard error is based on the sampled data and does not reflect the true sampling error of systematic errors from the forces in the MD or errors in the calculated EFG tensors.

#### 4.2.6 Extreme Narrowing Regime

A final issue we wish to address is the EN condition. EN manifests when the ACF $f_{2,m}$ decays rapidly compared to the Larmor precession period. Under this condition, the Fourier half‐transforms determining the spectral densities $g_{2,m}$ approach the time integral of $f_{2,m}$. This marked decay indicates a rapid spatial motion of the environment surrounding the analyte nucleus. Also, it may have its origin in fast changes in the molecular electronic structure caused by vibrational motions, contributing to the rapid loss of correlation of the EFG. Due to electrostatic interactions, ions of ILs move slower than small molecules in non‐ionic liquids. This particular condition can increase the correlation time of the EFG tensor components. To establish which effects prevail in EAN‐3D, $R_{1}$ and $R_{2}$ associated with an external field of 11.7 T were compared with those where $B_{0}=0$. The relaxation rates were determined as an isotropic average (option ForceIsotropy) of the EFG ACFs. These functions, in turn, were averaged over the 15 ions in each of the four independent trajectories. Under these conditions, the rates for $B_{0}=11.7$ T are $R_{1}=14.2\pm 1.1$ and $R_{2}=14.2\pm 1.1$ Hz, respectively, and, for $B_{0}=0$, both are equal to $R_{\mathrm{EN}}=14.2\pm 1.1$ Hz. Therefore, despite the ACFs extending over hundreds of picoseconds, the product $\omega_{0}\tau_{2,m}$ is very small for this system, characterizing the EN regime, and the rates determined explicitly for non‐EN conditions are the same within numerical errors. Consequently, the quadrupolar relaxation of EAN‐3D is essentially described by $\sigma_{\mathrm{iso}}$ and $\tau_{\mathrm{iso}}$, along with the nuclear spin quantum number and the nuclear quadrupolar cross section Q per Equation (7).

## 5 Conclusions

We implemented a methodology to simulate the spin relaxation of quadrupolar nuclei (I>12) in the deuterated ionic liquid ethylammonium nitrate (${\text{EtND}}_{3}{\text{NO}}_{3}$), based on relaxation theory combined with ab initio molecular dynamics to describe the temporal evolution of the system. Starting from simulations containing 15 cation‐anion pairs, the trajectories of the production steps were sampled. Each cation was extracted and surrounded by its closest neighbors, comprising the first two coordination spheres in the liquid. Calculations of these clusters were used to determine the autocorrelation functions of the electric field gradient tensors, from which the 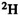 relaxation rates were determined. The result was close to the experimental rate by the standards of MD‐based spin relaxation rate calculations, with a rate of approximately 14 Hz, compared to an experimental value of 8 Hz.

The structure of the ionic liquid was evaluated via radial pair distribution functions. The peak positions of the RDFs agree relatively well with the values determined by neutron diffraction, particularly those associated with pairs of atoms belonging to the cation or involving the cation‐anion pair. The self‐diffusion coefficients of the cation and anion were estimated from the averaged squared displacements of the center of mass of the ions. Both diffusion coefficients were underestimated, with the cation coefficient agreeing reasonably well with the measured value. We attribute the differences observed between calculated and measured properties to the limitation of the GGA approximation in the ab initio molecular dynamics, which may be over‐structuring the liquid similar to what has been reported for liquid water.

The electric field gradients along the trajectories were calculated using electronic structure methods that do not feature periodic boundary conditions. For this reason, the procedure to mimic the liquid environment was to explicitly and implicitly solvate the cations. Thus, the first two surrounding shells, characterized by 13 nearest ions, enfold each cation, and the resulting 14‐ion system is additionally solvated using the SMD model. Tests have shown that relaxation rates obtained using only the first layer, containing five neighbors, differ by no more than 4% from the results from the two‐layer solvation, which is an insignificant difference when considering the overall deviation of the calculated rate from experiment. Despite this, we chose to work with two layers in the EFG calculations, which substantially increased the number of atoms in the EFG calculations but represented the environment around the cation in a more realistic way. Alternative methods to reduce the computational cost of this step, based on machine learning, have been reported recently [28].

Seven different basis set and functional combinations were tested for calculating the EFGs, aiming to assess the effect of the exchange‐correlation functional or diffuse basis functions, and the effects from including the VV10 dispersion functional self‐consistently. The relaxation properties of EAN‐3D were observed to be susceptible to the addition of the VV10 correction, although the effect was not dramatic. Our main finding for EAN‐3D is that the autocorrelation functions and the resulting relaxation rates are not converged until the simulations extend to at least 300 ps (based on 15 cations in each of four independent trajectories), and longer simulations are required to ascertain convergence barring very rare events. The long required simulation times are tentatively attributed to a relatively slow movement of the ions in the ionic liquid as well as comparatively strong electrostatic interactions within the system, which requires several hundred picoseconds to eliminate the time‐correlation in the electric field gradient tensor components. Given the EFG correlation times of two or three dozen picoseconds, the trajectory lengths of more than 600 ps would appear to be more than sufficient for relaxation rate studies, although it is worth noting that convergence in the longitudinal relaxation rate was obtained only for the average of the four independent trajectories, not for the trajectories individually.

The longitudinal relaxation rate determined for EAN‐3D in this study is 13.3±1.0 Hz, which is about 60% above the experimental value. As discussed in previous literature on the calculation of quadrupolar relaxation in different systems, such a result is quite satisfactory for the applied level of theory. Finally, the quadrupolar relaxation in EAN occurs within the extreme narrowing condition. Therefore, the longitudinal and transverse relaxation rates are proportional to the variance of the electric field gradient and the associated correlation time. The present study also highlights again the benefits of multiple independent trajectories for the study of complex systems.

## Funding

This work was supported by Fundação de Amparo à Pesquisa do Estado de São Paulo (2024/17236‐1), Coordenação de Aperfeiçoamento de Pessoal de Nível Superior, Finance Code 001, Conselho Nacional de Desenvolvimento Científico e Tecnológico (304653/2023‐3), and National Science Foundation (CHE‐2503332).

## Conflicts of Interest

The authors declare no conflicts of interest.

## Supporting information


**Data S1:** Supplementary Information.

## Data Availability

Supporting Information includes: (i) Plots of the radial distribution functions of each atom pair listed in Table 1 (Figures S1 and S2), (ii) Spectral densities of deuterium computed for $B_{0}$ within the interval $\left[0,100\right]$ T (Figure S3), (iii) Plots showing the behavior of the longitudinal relaxation rate as a function of the trajectory duration for the four independent trajectories (Figure S4), (iv) The EFG autocorrelation functions of cations two and eight of EAN‐3D trajectory no. one (Figure S5), and (v) A theoretical description of the autocorrelation function determined via the Wiener‐Khinchin theorem combined with the discrete Fourier transform.
