# Supplementary material for: Ab Initio Molecular Dynamics Study of Quadrupolar Spin Relaxation in an Ionic Liquid
Source: J Comput Chem. 2026 Jan 24;47(3):e70311. doi: 10.1002/jcc.70311 (PMC12831640; doi:10.1002/jcc.70311)
Supplement: Supplementary file 1 — Data S1: Supplementary Information. [file JCC-47-0-s001.pdf]

Supporting Information for: 'Ab initio molecular dynamics study of quadrupolar spin relaxation in an ionic liquid'

Luciano Nassif Vidal[ab,*], Lucas Colucci Ducati[b,*], Jochen Autschbach[c,*]

[a]Departamento de Química e Biologia
Universidade Tecnológica Federal do Paraná
Curitiba, PR, 81280-340, BR
email: lnvidal@utfpr.edu.br

[b]Department of Fundamental Chemistry
Institute of Chemistry
University of Sao Paulo
Sao Paulo, SP, 05508-000, BR
email: ducati@iq.usp.br

[c]Department of Chemistry
University at Buffalo
State University of New York
Buffalo, NY 14260-3000, USA
email: jochena@buffalo.edu

January 14, 2026

Sections included in this document:

S1 Radial distribution functions, EFG autocorrelation functions, Spectral Densities, and Relaxation Rates **S2**

S2 Autocorrelation function from Fourier Transform **S6**

S2.1 Discrete Fourier Transform S7
S2.2 Padding with zeros S8
S2.3 Case Study: (a) No addition of zeros, $N_p = N$ S9
S2.4 Case Study: (b) Addition of N zeros, $N_p = 2N$ S10

S1 Radial distribution functions, EFG autocorrelation functions, Spectral Densities, and Relaxation Rates

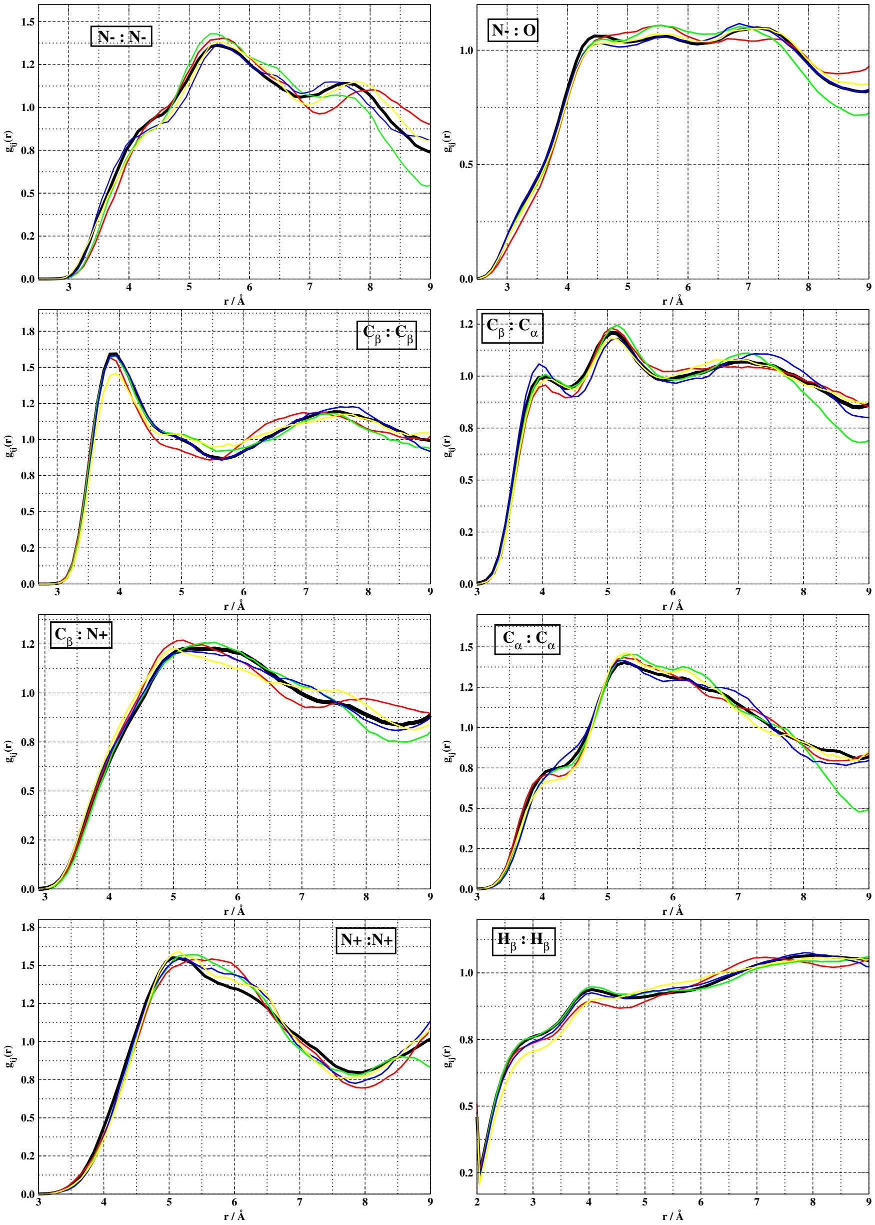

Figure S1: Radial distribution functions for anion-anion and cation-cation atom-pairs for the four aiMD trajectories. The thicker black line corresponds to the average RDF.

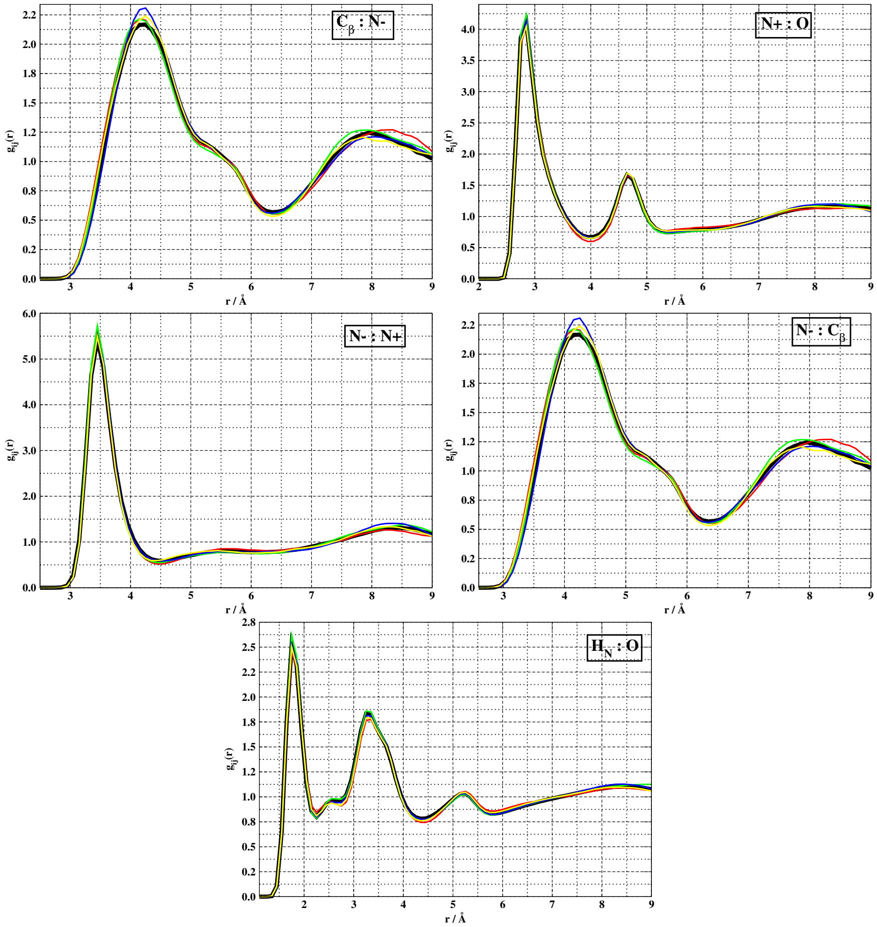

Figure S2: Radial distribution functions for cation-anion atom-pairs for the four aiMD trajectories. The thicker black line corresponds to the average RDF.

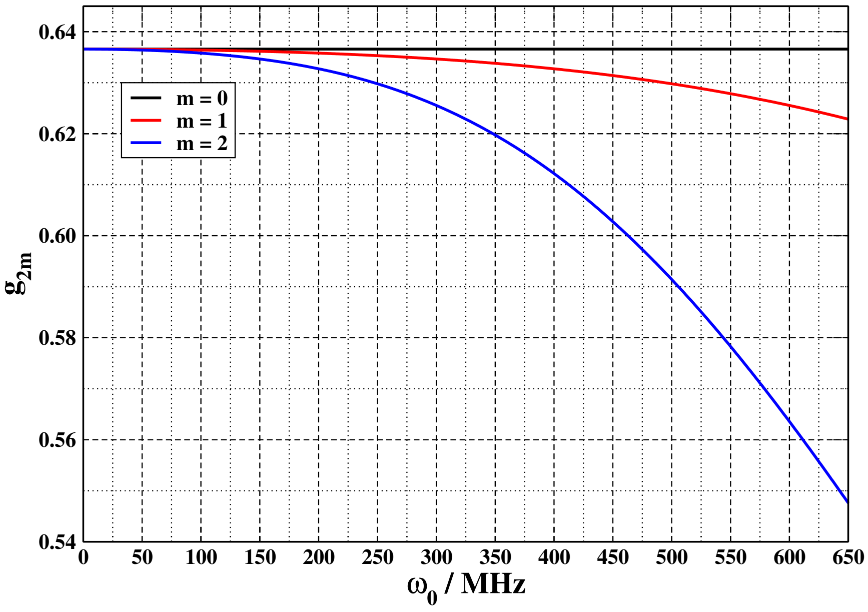

Figure S3: Spectral densities, $g_{2,m}$ (in $E_h^2 \cdot e^{-2} \cdot a_0^{-4} \cdot ps$), computed from an isotropic autocorrelation function (`ForceIsotropy` option), as a function of the Larmor frequency of deuterium, ω_0 ($0 \leq B_0 \leq 100$ T). The autocorrelation function used to generate $g_{2,m}$ is an average of 60 results, i.e., 15 ACFs for each independent trajectory.

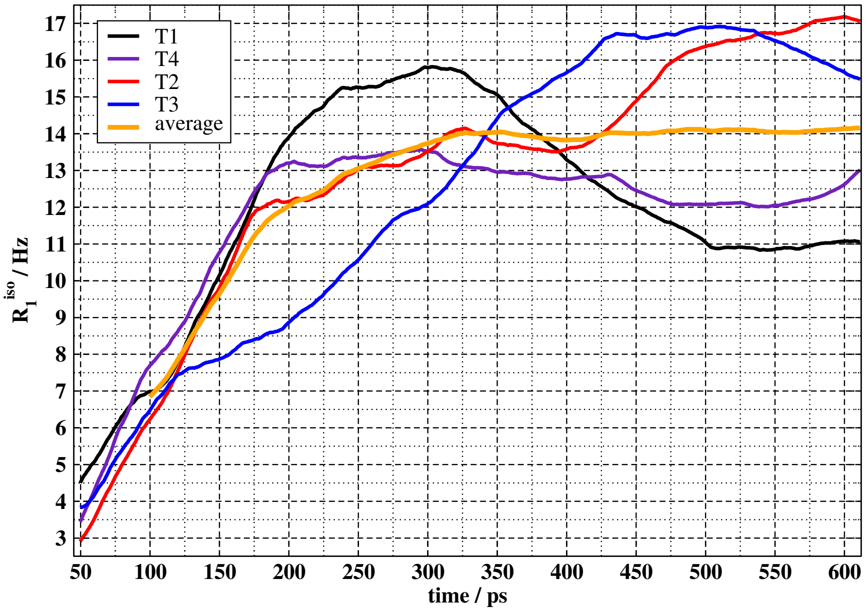

Figure S4: Isotropic longitudinal relaxation rates ($B_0 = 11.7$ T) computed for the four independent trajectories, as a function of the duration of the production stage.

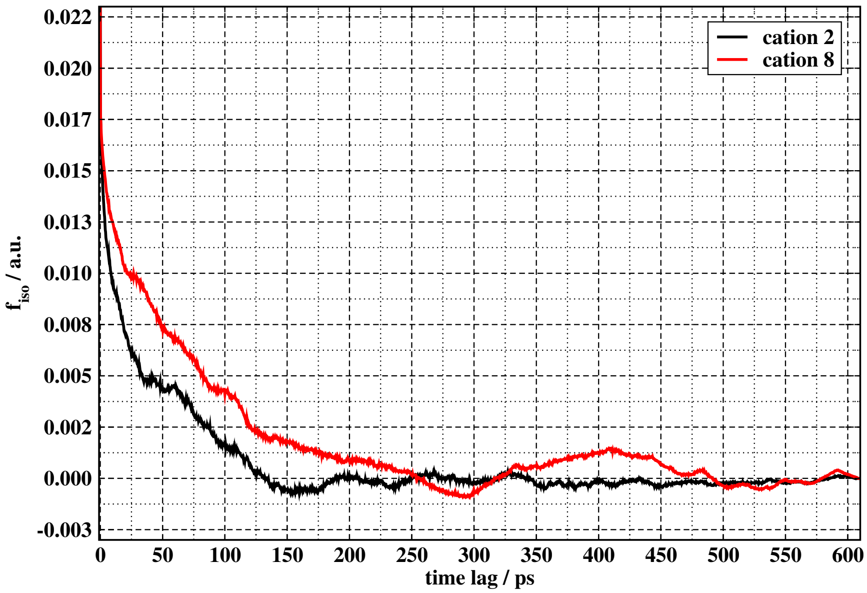

Figure S5: EFG isotropic autocorrelation functions for cations two and eigth using trajectory no. one.

S2 Autocorrelation function from Fourier Transform

The Fourier transform of the time function $f(t)$ is defined as follows:[1]

$$F(\nu) \equiv \int_{-\infty}^{+\infty} f(t)e^{2\pi i\nu t}dt. \tag{S1}$$

The Fourier transform $F(\nu)$ is a function of frequency "ν" and has the dimension of the function "f" multiplied by time. The inverse transform is given by:

$$f(t) = \int_{-\infty}^{+\infty} F(\nu)e^{-2\pi i\nu t}d\nu. \tag{S2}$$

In ergodic processes, a time average can replace the ensemble average of a stochastic function. Under these circumstances, the autocorrelation function (ACF) is given by the limit:

$$C(\tau) = \lim_{T\to\infty} \frac{1}{(T-t_0)} \int_{t_0}^{T} f(t)^* f(t+\tau)dt, \tag{S3}$$

where τ is the *time lag* and t_0 an arbitrary time origin (usually zero). Using the Fourier integral theorem to express the function $f(t+\tau)$:

$$\begin{aligned} f(t+\tau) &= \int_{-\infty}^{+\infty} e^{-2\pi i\nu(t+\tau)}d\nu \int_{-\infty}^{+\infty} f(t')e^{2\pi i\nu t'}dt' \\ &= \int_{-\infty}^{+\infty} F(\nu)e^{-2\pi i\nu(t+\tau)}d\nu. \end{aligned}$$

Replacing $f(t+\tau)$ in Eq. (S3) with the above result, we obtain:

$$\begin{aligned} C(\tau) &= \lim_{T\to\infty} \frac{1}{(T-t_0)} \int_{t_0}^{T} f(t)^* \left[\int_{-\infty}^{+\infty} F(\nu)e^{-2\pi i\nu(t+\tau)}d\nu\right] dt \\ &= \lim_{T\to\infty} \frac{1}{(T-t_0)} \int_{-\infty}^{+\infty}\int_{t_0}^{T} f(t)^* F(\nu)e^{-2\pi i\nu t}e^{-2\pi i\nu\tau}dt\, d\nu \\ &= \lim_{T\to\infty} \frac{1}{(T-t_0)} \int_{-\infty}^{+\infty} F(\nu)e^{-2\pi i\nu\tau}\left[\int_{t_0}^{T} f(t)^* e^{-2\pi i\nu t}dt\right] d\nu. \end{aligned}$$

By replacing t_0 with the limit of $t_0 \to \infty$, the term in the square parentheses above will become the complex conjugate of F(ν) when both limits have been taken:

$$\begin{aligned} C(\tau) &= \lim_{\substack{t_f\to\infty \\ t_i\to-\infty}} \left(\frac{1}{t_f - t_i}\right) \int_{-\infty}^{+\infty} F(\nu)e^{-2\pi i\nu\tau} \int_{t_i}^{t_f} f(t)^* e^{-2\pi i\nu t}dt\, d\nu \\ &= \lim_{\substack{t_f\to\infty \\ t_i\to-\infty}} \left(\frac{1}{t_f - t_i}\right) \int_{-\infty}^{+\infty} F(\nu)F(\nu)^* e^{-2\pi i\nu\tau}d\nu. \end{aligned} \tag{S4}$$

Here, T was replaced with t_f and t_0 with t_i. The last integral above, in the variable ν, represents the inverse transform of $|F(\nu)|^2$. This result is known as the Wiener-Khinchin theorem. Therefore, an approximation to the autocorrelation function is obtained by doing:

$$C(\tau) \approx \frac{1}{(t_f - t_i)} \int_{-\infty}^{+\infty} |F(\nu)|^2 e^{-2\pi i \nu \tau} d\nu,$$

with $(t_f - t_i)$ being the length of the molecular dynamics trajectory.

S2.1 Discrete Fourier Transform

The discretized form of the Fourier transform of $f(t)$ is written as:[2]

$$F(\nu_n) = \Delta t F_n, \tag{S5}$$

where Δt is a regular time interval that separates N consecutive values of the sampled function, and F_n is defined by:

$$F_n \equiv \sum_{k=0}^{N-1} f_k \, e^{2\pi i \nu_n t_k}, \tag{S6}$$

in which:

$$\begin{aligned} t_k &\equiv k\Delta t, & k &= 0, 1, 2, \cdots, N-1 \\ f_k &\equiv f(t_k) & & \\ \nu_n &\equiv \frac{n}{N\Delta t}, & n &= -\frac{N}{2}, -\frac{(N-1)}{2}, \cdots, -\frac{1}{2}, 0, \frac{1}{2}, \cdots, \frac{(N-1)}{2}, \frac{N}{2}. \end{aligned} \tag{S7}$$

In Definition (S7), t_k and ν_n are the times and frequencies of the discretized Fourier transform, respectively. In the definition of frequencies ν_n, it is implicit that N must be even. From Definition (S7), the function F_n can be written as:

$$F_n = \sum_{k=0}^{N-1} f_k \, e^{2\pi i k n / N}.$$

The discretized form of the inverse transform is shown below:

$$f_k = \frac{1}{N\Delta t} \sum_{n=0}^{N-1} (\Delta t \, F_n) e^{-2\pi i k n / N}.$$

Therefore, the discretized form of the autocorrelation function is equal to:

$$\begin{aligned} C_k &= \frac{1}{(N-1)\Delta t} \left[\frac{1}{N\Delta t} \sum_{n=0}^{N-1} |\Delta t F_n|^2 e^{-2\pi i n k / N} \right] \\ &= \frac{1}{N(N-1)} \sum_{n=0}^{N-1} |F_n|^2 \, e^{-2\pi i n k / N}. \end{aligned} \tag{S8}$$

In the QRELAX implementation, $t_i = 0$ and $t_f = (N-1)\Delta t$, justifying the appearance of the denominator "$(N-1)\Delta t$" in Eq. (S8).

S2.2 Padding with zeros

When the function $f(t)$ is padded with zeros, so that $f_k = 0$ for $k > N$, and $k_{\max} = N_p$, the new frequencies at which the Fourier transform is calculated are:

$$\nu_n = \frac{n}{N_p \Delta t}, \qquad n = -\frac{N_p}{2}, -\frac{(N_p-1)}{2}, \cdots, -\frac{N}{2}, \cdots, 0, \cdots, \frac{N}{2}, \cdots, \frac{(N_p-1)}{2}, \frac{N_p}{2}.$$

Now, the F_n are calculated at different frequencies than in the previous case (no padding) due to the appearance of the divisor N_p in the complex exponential, i.e.:

$$\begin{aligned} F_n &= \sum_{k=0}^{N_p-1} f_k\, e^{2\pi i k n/N_p} \\ &= \sum_{k=0}^{N-1} f_k\, e^{2\pi i k n/N_p}. \end{aligned}$$

As before, we have only N values of $f(t)$ contributing to F_n. Furthermore, the value of F_n at the maximum frequency does not change since it depends only on the sampling rate Δt.

Let us write the expression corresponding to the square of the Fourier transform and the corresponding expression for the autocorrelation function:

$$\begin{aligned} F_n F_n^* &= \sum_{l=0}^{N_p-1} \sum_{l'=0}^{N_p-1} f_l f_{l'}^*\, e^{2\pi i(l-l')n/N_p} \\ C_k &= \frac{1}{N_p(N-1)} \sum_{n=0}^{N_p-1} \sum_{l=0}^{N_p-1} \sum_{l'=0}^{N_p-1} f_l f_{l'}^*\, e^{2\pi i(l-l'-k)n/N_p} \\ &= \frac{1}{N_p(N-1)} \sum_{l=0}^{N_p-1} \sum_{l'=0}^{N_p-1} f_l f_{l'}^* \left(\sum_{n=0}^{N_p-1} e^{2\pi i(l-l'-k)n/N_p} \right). \end{aligned}$$

Using the orthogonality relation,

$$\sum_{c=0}^{M-1} e^{2\pi i(a-b)c/M} = M\left(\delta_{a,b} + \delta_{b-a,M}\right),$$

where a, b and c are integers and M is even, the autocorrelation function becomes equal to:

$$C_k = \frac{1}{(N-1)} \sum_{l=0}^{N_p-1} \sum_{l'=0}^{N_p-1} f_l f_{l'}^* \left(\delta_{l-l',k} + \delta_{k+l'-l,N_p}\right).$$

Since $f_l = 0$ if $l > N$, we can still write:

$$C_k = \frac{1}{(N-1)} \sum_{l=0}^{N-1} \sum_{l'=0}^{N-1} f_l f_{l'}^* \left(\delta_{l-l',k} + \delta_{k+l'-l,N_p}\right).$$

The above result leads to the following general expression for calculating the autocorrelation function:

$$C_k = \frac{1}{(N-1)}\left(\sum_{l=k}^{N-1} f_l f_{l-k}^* + \sum_{k'=1}^{k} f_{k-k'} f_{N_p-k'}^*\right). \tag{S9}$$

The first term, involving the product $f_l f_{l-k}^*$, is as expected for an autocorrelation function since, for $\tau = t_0 = 0$, we have a sum of terms of the type $f_l f_l^*$, while for $\tau = t_1 = \Delta t$, the sum involves the product $f_l f_{l-1}^*$ and so on. However, we cannot attribute physical meaning to the second sum, containing the product $f_{k-k'} f_{N_p-k'}^*$.

S2.3 Case Study: (a) No addition of zeros, $N_p = N$

When $N_p = N$, that is, there is no addition of zeros in the function f_k, the values obtained for the autocorrelation function at different times are given by:

$$\begin{aligned}
C_0 &= \frac{1}{(N-1)}\sum_{l=0}^{N-1} |f_l|^2 \\
C_1 &= \frac{1}{(N-1)}\left[\left(\sum_{l=1}^{N-1} f_l f_{l-1}^*\right) + f_0 f_{N-1}^*\right] \\
C_2 &= \frac{1}{(N-1)}\left(\sum_{l=2}^{N-1} f_l f_{l-2}^* + \sum_{k'=1}^{2} f_{2-k'} f_{N-k'}^*\right) \\
&\vdots \\
C_{\frac{N}{2}-1} &= \frac{1}{(N-1)}\left(\sum_{l=\frac{N}{2}-1}^{N-1} f_l f_{l-(\frac{N}{2}-1)}^* + \sum_{k'=1}^{\frac{N}{2}-1} f_{(\frac{N}{2}-1)-k'} f_{N-k'}^*\right) \\
C_{\frac{N}{2}} &= \frac{1}{(N-1)}\left(\sum_{l=\frac{N}{2}}^{N-1} f_l f_{l-\frac{N}{2}}^* + \sum_{k'=1}^{\frac{N}{2}} f_{\frac{N}{2}-k'} f_{N-k'}^*\right) \\
C_{\frac{N}{2}+1} &= C_{\frac{N}{2}-1}^* \\
C_{N-2} &= C_2^* \\
C_{N-1} &= C_1^*.
\end{aligned}$$

Therefore, considering only the absolute values of C_k, we have only $N/2$ distinct values for the autocorrelation function.

S2.4 Case Study: (b) Addition of N zeros, $N_p = 2N$

In this situation, N values of $f(t)$ were sampled, corresponding to f_k with $0 \leq k \leq N-1$. The other $N-1$ to $2N-1$ values of $f(t)$ were completed with zeros. The autocorrelation function, in this case, does not contain the non-physical term given by $\sum_{k'} f_{k-k'} f^*_{N_p-k'}$. It is shown the ACF in different times below:

$$
\begin{aligned}
C(t_0) &= \frac{1}{(N-1)} \sum_{l=0}^{N-1} |f_l|^2 \\
C(t_1) &= \frac{1}{(N-1)} \sum_{l=1}^{N-1} f_l f^*_{l-1} \\
C(t_2) &= \frac{1}{(N-1)} \sum_{l=2}^{N-1} f_l f^*_{l-2} \\
&\vdots \\
C(t_{N-1}) &= \frac{1}{(N-1)} f_{N-1} f^*_0 \\
C(t_N) &= 0 \\
C(t_{N+1}) &= C(t_{N-1})^* \\
&\vdots \\
C(t_{2N-1}) &= C(t_1)^*.
\end{aligned}
$$

References

[1] Arfken, G. B.; Weber, H. J. *Mathematical Methods for Physicists;* Academic Press: San Diego, 4th ed.; 1995.

[2] Press, W. H.; Teukolsky, S. A.; Vetterling, W. T.; Flannery, B. P. *Numerical Recipes: The Art of Scientific Computing;* Cambridge University Press: New York, NY, 2nd ed.; 1997.
